# The Mountain Region of Western Texas as a Health Resort

**Published:** 1881-03

**Authors:** F. Charles Lawrence

**Affiliations:** Kerrville, Texas


### ARTICLE IX.

### THE MOUNTAIN REGION OF WESTERN TEXAS AS A HEALTH RESORT. By F. Charles Lawrence, A.M., M.D., Kerrville, Texas.

Since the completion of the "Sunset" and International Railways to San Antonio, Southwestern Texas is becoming quite a favorite locality for Western and Northern physicians to send their patients for the winter months, whose condition may require a removal from the inclemency of a Northern winter.

The great majority of invalids remain in San Antonio, having been directed there by their medical adviser, and with all due respect to our learned *confreres* of the North, it would be no exaggeration to state that not more than one in twenty have a personal knowledge of the locality, beyond the reading of an article in some periodical, written by persons who are incapable of estimating the merits of a climate as regards its health-restoring properties.

The leading authorities on phthisis, both American and foreign, advocate, as the most essential qualities of any climate favoring the retardation and cure of this disease, the following climatic conditions, viz.:

A moderate altitude above sea level, between 1,200 and 2,000

feet, with a moderate rain-fall and freedom from malarial influences. Under this heading may very appropriately be considered the coexistence, with the qualifications just mentioned, of a light, porous subsoil, favoring rapid drainage after rain. *Vide* Buchanan and Bowditch.

A winter temperature sufficiently mild to admit, without discomfort, daily exercise in the open air.

Nutritious, well-cooked food, and properly ventilated sleeping rooms.

Now, San Antonio is located in a valley only 700 feet above the sea, and the atmosphere is more or less highly charged with moisture from the San Antonio river and San Pedro creek, which meander through the town, besides the water that is carried through most of the streets in irrigating ditches. The subsoil is of a clayey character, and during rainy weather its streets are well-nigh impassable for mud, while during the dry season the dust renders life almost intolerable.

Some of the San Antonio physicians send their patients to Boerne, a small village thirty miles northwest of the city; but its location on a substratum of clay, and exposed to the damp south winds which invariably succeed the "Northers," together with its water supply from the Cibolo Creek, which contains a considerable amount of organic matter in solution, renders this point very undesirable in many respects as a winter residence.

Malarial fever of a severe type prevailed there during the months of last autumn, and during a two week's sojourn at the Boerne Hotel, on the banks of the Cibolo, I suffered from a severe attack, only recovering after going up to the mountains.

Thirty-six miles northwest of Boerne, in the midst of beautiful mountain scenery, is the village of Kerrville, the county seat of Kerr county. Its elevation is 1,800 feet above the sea, and possessing so many advantages on the score of health wanting in San Antonio, I take the liberty of placing a description of it before the profession.

Kerrville is located on the banks of the Guadalupe river; whose water is very free from organic matter, and so clear that objects on its bottom may be seen at a great depth. The town rests on a gravelly substratum, and the drainage is so perfect that even after a heavy rain storm, a few hours' sunshine renders the earth perfectly dry. A high range of hills to the northward breaks the force of the "Northers," while the distance from the sea—over 250 miles—deprives the returning south winds of much of their moisture and rawness.

The winters are very mild, snow and ice, in ordinary seasons, being almost unknown, and the winter sometimes passing without any rain, the invalid may, with the exception of a few days about Christmas, take daily exercise in a balmy and invigorating mountain air.

Good carriage roads extend in various directions, and fine fishing and hunting abound in the vicinity. At the headwaters of the Guadalupe several caves invite the curious to seek for "hidden mysteries."

The people are very kind and hospitable, being persons of high intelligence and culture, who came here in the first and second stages of phthisis and have been completely restored to health.

An additional advantage is now offered by this place, and one not be overlooked by the invalid, viz.: good hotel accommodations and at a moderate price. Mr. J. F. H. Back, of Cincinnati, has recently purchased the hotel in this town, and one can secure, what every one knows who travels in Texas, that *rare luxury*—well-cooked food and clean, comfortable rooms.

During the summer months the heat is never excessive, and with a constant southern breeze and cool nights, the summer can be passed much more pleasantly than at the North. During at least eight months in the year one can sleep in the open air with not only impunity, but in the early stages of phthisis with positive benefit. Invalids who propose coming to this mountain region will find a daily line of comfortable hacks running from San Antonio to Kerrville and the upper country. To any physicians who may desire further information concerning the climate of the country, I will here state that Dr. Petersen, of Comfort, Texas, who has been for some time past a voluntary observer for the U. S. Signal Service in this region, will very gladly answer any inquiries that may be addressed to him on the subject.

